# How I Do It: Perioperative Use of Micro-Axial Pumps in High Risk Coronary Artery Bypass Grafting: The Early Johns Hopkins Experience

**DOI:** 10.3390/jcdd13050193

**Published:** 2026-04-30

**Authors:** Salman Zaheer, Mohammad Aref, Oldrich Virag, Bogdan Ivanov, Chetan Pasrija, Antonio Polanco, Hamza Aziz, Ahmet Kilic

**Affiliations:** 1 Division of Cardiac Surgery, Penn State Milton S. Hershey Medical Center, 200 Campus Dr., Hershey, PA 17033, USA; 2 Division of Cardiac Surgery, Johns Hopkins Hospital, Baltimore, MD 21287, USA; maref2@jhmi.edu (M.A.); cpasrija@jhmi.edu (C.P.); apolonco2@jh.edu (A.P.); haziz2@jhmi.edu (H.A.); akilic2@jhmi.edu (A.K.); 3 Penn State College of Medicine, 700 HMC Crescent Rd, Hershey, PA 17033, USA; ovirag@pennstatehealth.psu.edu (O.V.); bivanov@pennstatehealth.psu.edu (B.I.)

**Keywords:** mechanical circulatory support, Impella 5.5, postcardiotomy shock, cardiac surgery, left ventricular dysfunction, perioperative support, microaxial pump

## Abstract

Patients with left ventricular dysfunction undergoing cardiac surgery face a heightened risk of perioperative complications, including postcardiotomy shock (PCS). Conventional management with inotropes and vasopressors can exacerbate end-organ dysfunction, underscoring the need for alternative strategies. The planned use of mechanical circulatory support (MCS) devices, such as the Impella, offers a proactive approach to mitigating PCS in high-risk patients. This study presents our early experience at Johns Hopkins with planned Impella utilization in high-risk cardiac surgery. We detail our risk stratification methodology, patient selection criteria, and perioperative management strategies. Our proposed risk stratification scoring system incorporates surgical intent, preoperative myocardial function, anticipated postoperative course, and exit strategy to identify optimal candidates for perioperative MCS. We describe the intraoperative central placement technique for the Impella 5.5, perioperative management protocols—including anticoagulation strategies and weaning protocols—and postoperative device extraction. A retrospective review of our first 11 consecutive patients with severely reduced left ventricular ejection fraction (<30%) who underwent Impella-assisted cardiac surgery demonstrated favorable outcomes, with no postoperative mortality and a two-year follow-up. Our findings suggest that planned Impella use in high-risk cardiac surgery is both feasible and beneficial. However, further studies are necessary to validate these results, assess long-term outcomes, and evaluate cost-effectiveness.

## 1. Introduction

The incidence of postcardiotomy shock (PCS) has increased over the last two decades, likely due to an aging population requiring cardiac surgery and a higher baseline morbidity [1]. The incidence of PCS sits between 0.5 and 6% of cases, depending on the definition and patient population, but in-hospital mortality rates remain high at approximately 67% [1,2]. Patients with severely reduced left ventricular ejection fraction (LVEF), defined as LVEF < 30%, are a particularly vulnerable cohort with a substantially elevated risk for postoperative low cardiac output syndrome and cardiogenic shock [3,4].

Management of PCS traditionally relies on escalating inotropic and vasopressor support. However, this can increase myocardial oxygen demand and worsen end-organ dysfunction [1]. Mechanical circulatory support (MCS) is often required when pharmacological support is inadequate. However, delayed MCS initiation after shock has already developed is associated with poor outcomes, as preserving end-organ function, especially renal function, is critical for survival [3,5].

Popular MCS devices have included intra-aortic balloon pumps (IABPs), which provide limited hemodynamic support, or venoarterial extracorporeal membrane oxygenation (VA-ECMO), which increases left ventricular (LV) afterload and can cause ventricular distension. However, the introduction of microaxial flow pumps such as the Impella device family changed the landscape of temporary MCS. The Impella provides direct LV unloading while maintaining antegrade flow up to 5.5 L/min [6,7,8]. Recent evidence shows that prophylactic Impella placement in high-risk cardiac surgery patients may improve outcomes compared to reactive strategies [9,10].

Prophylactic Impella use in patients with LVEF < 30% undergoing cardiac surgery has shown 30-day survival rates of 80–93% and significant postoperative improvement in ejection fraction [9,10]. However, all studies are observational and predominantly retrospective, and Impella use is associated with significant complications including bleeding (32.8%), vascular complications (15.8%), hemolysis (5–42%), and acute kidney injury (35–50%) [11,12,13].

Further research is needed on optimal MCS device selection. Compared to IABP, Impella provides more robust hemodynamic support but with higher complication rates [11,14]. Compared to VA-ECMO, Impella avoids the complications of LV distension and differential hypoxia but provides no respiratory support [8,15]. The American Association for Thoracic Surgery acknowledges that axial-flow devices like Impella “drastically reduce LV end-diastolic pressure and volume and may be better poised to support systemic perfusion while allowing the heart to recover” compared to IABP, but there are sparse prospective randomized trials in the realm of cardiac surgery [7].

This study presents our early experience with planned, prophylactic Impella 5.5 utilization in high-risk cardiac surgery patients at Johns Hopkins. We detail our risk stratification methodology, patient selection criteria, intraoperative central placement technique, and comprehensive perioperative management protocols. Our retrospective review of 11 consecutive patients with severely reduced LVEF (<30%) demonstrated no postoperative mortality and favorable outcomes at two-year follow-up. These findings suggest that a proactive, protocol-driven approach to MCS in carefully selected high-risk patients may improve outcomes, though further prospective studies are necessary to further validate these findings.

## 2. Materials and Methods

### 2.1. Unique Properties of Microaxial Pump Compared to Other MCS

The prophylactic use of temporary MCS at the time of surgery in patients with ischemic heart disease and LV dysfunction unloads the ventricle, supports systemic circulation, and reduces the need for inotropes and vasopressors to maintain end-organ perfusion. Various types of MCS exist, each with distinct hemodynamic effects. In this section, we focus on the Impella device and detail the rationale behind its selection for prophylactic temporary MCS in high-risk cardiac surgery.

**Figure 1:**
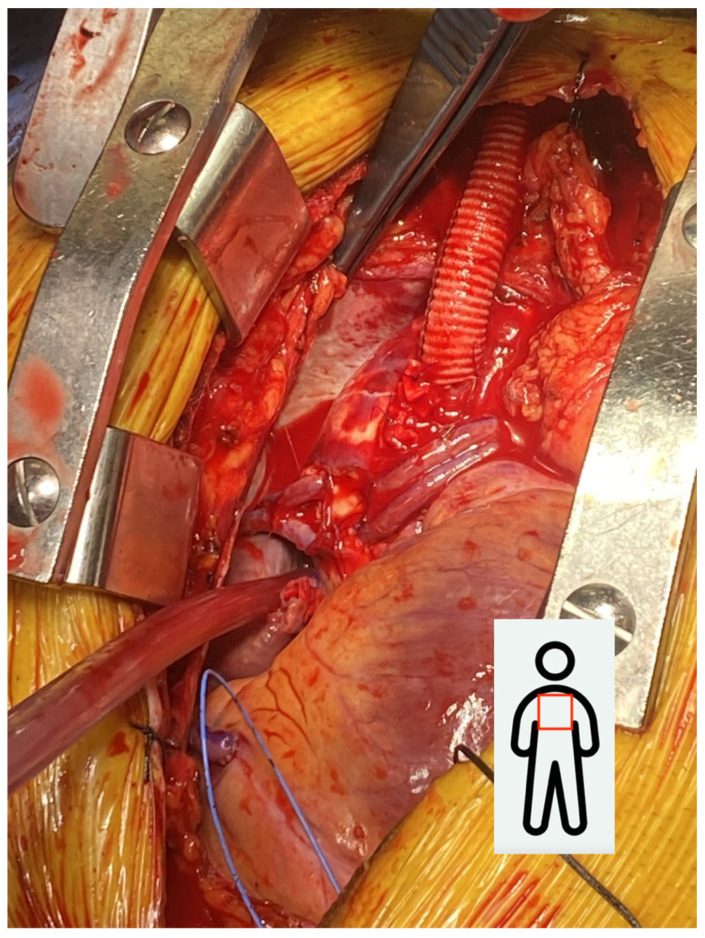
10 mm Dacron graft sewn in the ascending aorta.

**Figure 2:**
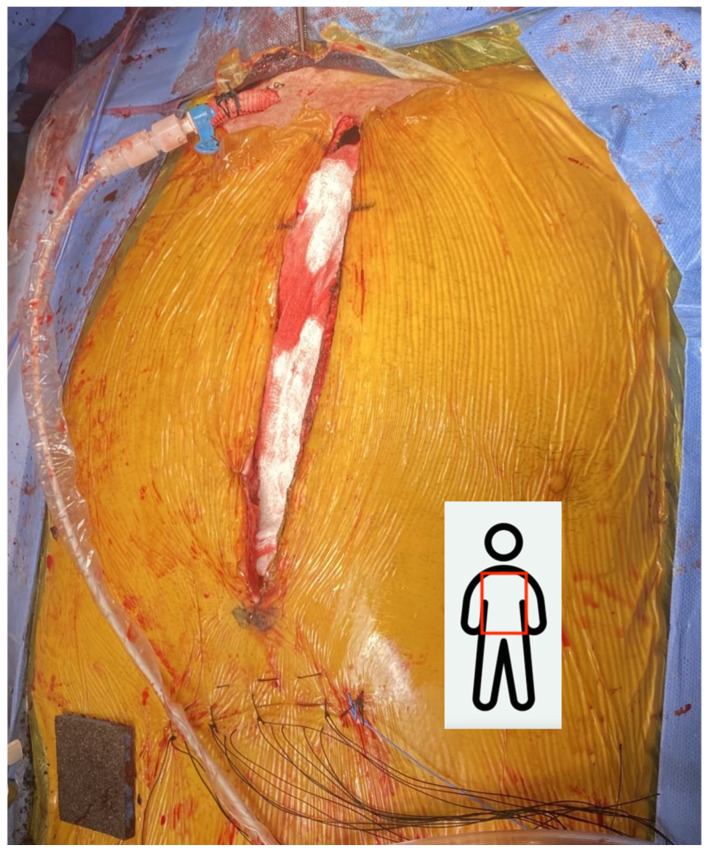
10 mm Dacron graft tunneled to the supraclavicular incision with the Impella in place.

The Impella (Abiomed, Danvers, MA, USA) is a catheter-based, transaortic microaxial pump that actively unloads the LV by moving blood from the LV into the ascending aorta (Figure 1 and Figure 2). The Impella provides up to 6 L/min of cardiac output augmentation, making it a valuable tool for perioperative hemodynamic support. The Impella 5.5, FDA-approved in 2019, is the largest of the Impella microaxial pumps (19Fr pump, 21Fr cannula) and is capable of delivering a maximum flow of 5.5–6 L/min via axillary or central cannulation.

Compared to other MCS devices, the Impella offers several advantages for temporary prophylactic support during cardiac surgery. While the IABP is commonly used for its ease of insertion, availability, and lower cost, its limitations are evident in patients with significant LV dysfunction. The IABP reduces systolic afterload and enhances diastolic coronary perfusion but provides limited augmentation of cardiac output and insufficient LV unloading. In contrast, the Impella actively unloads the LV while simultaneously increasing cardiac output, offering superior hemodynamic support. This feature particularly benefits patients with compromised ventricular function, where IABP efficacy is limited.

The Impella’s benefits include its high maximal flow capacity (5.5–6 L/min) and the ability to be placed via axillary or central cannulation. This placement facilitates early postoperative ambulation compared to femoral access. However, successful Impella support requires a functioning right ventricle, necessitating vigilant monitoring of right ventricular performance. Potential drawbacks of the Impella 5.5, such as its larger size and the need for open axillary or central cannulation, are mitigated when the device is placed prophylactically during surgery.

Contraindications to Impella use include the presence of a mechanical aortic valve and LV thrombus, as outlined in the device’s clinical guidelines. Additionally, caution is advised in patients with significant aortic valve stenosis or severe peripheral arterial disease. Despite these limitations, the Impella 5.5 remains a highly effective MCS option for planned perioperative support in high-risk cardiac surgery, offering robust hemodynamic stability and improved postoperative outcomes.

### 2.2. Patient Selection for Perioperative MCS Based on Risk

Similarly to the approach in protected percutaneous coronary intervention (PCI), the utilization of perioperative MCS in cardiac surgery requires careful consideration of the associated risks, costs, and potential benefits. In addition to the potential complications related to Impella placement and support, the device itself represents a significant financial investment. Therefore, planned perioperative use of the Impella must be strategically targeted to patients most likely to benefit from its hemodynamic support.

In this section, we outline our systematic approach for identifying patients at heightened risk for PCS who may derive the greatest benefit from perioperative MCS. Our patient selection framework is based on four key factors: intent of operation, preoperative myocardial function and reserve, anticipated postoperative course, and the ability to implement a rescue or exit strategy if the patient cannot be weaned from temporary MCS.

Table 1 summarizes these factors and presents a scoring system designed to guide the identification of patients most suitable for planned perioperative MCS. This scoring system facilitates objective decision-making regarding the implementation of Impella support in high-risk cardiac surgery patients.

In the present series, all 11 patients satisfied our institutional candidacy framework for planned perioperative MCS. Operative intent consisted of high-risk CABG-based procedures, including multivessel CABG with or without concomitant procedures. Preoperative myocardial dysfunction was substantial, with a mean LVEF of 17.5% and mean LVEDD of 6.14 cm. Patients were selected only when the anticipated postoperative course was judged to carry meaningful risk for postcardiotomy shock and when a clear rescue pathway was present, including the feasibility of escalation to advanced heart failure therapies or other definitive strategies if recovery did not occur. Patient-level operative, echocardiographic, and perioperative characteristics relevant to this selection framework are summarized in Table 2, Table 3, Table 4 and Table 5.

### 2.3. Intent of Operation

The risk of developing PCS and related complications varies depending on the type and complexity of the cardiac surgery performed. Procedures anticipated to be more complex and prolonged—such as coronary artery bypass graft (CABG) with three or more targets or CABG combined with valve repair—are associated with a higher risk of PCS compared to isolated CABG procedures involving one to two targets. Among concomitant valve surgeries, mitral valve procedures carry a particularly elevated risk of PCS, especially in the setting of LV dysfunction [16].

### 2.4. Preoperative Myocardial Function and Reserve

Assessment of preoperative myocardial function and reserve is critical for identifying patients who may struggle to tolerate the hemodynamic stress of cardiac surgery. Key factors include LVEF, LV cavity size, wall thickness, and the presence of regional wall motion abnormalities. Additionally, systolic blood pressure pulsatility serves as an indirect marker of myocardial contractile reserve, providing further insight into the patient’s preoperative cardiac function.

Preoperative right ventricular assessment is performed primarily by comprehensive echocardiography, including RV size and function, TAPSE, fractional area change, severity of tricuspid regurgitation, and estimated pulmonary pressures; in selected patients with uncertain RV reserve or concern for elevated pulmonary vascular load, right heart catheterization may provide additional useful hemodynamic information.

### 2.5. Anticipated Postoperative Course

Predicting the postoperative course involves evaluating potential intraoperative and perioperative challenges that may compromise recovery. Considerations include the anticipated vasoactive inotropic score (VIS), the complexity of the surgical procedure, and the presence of significant comorbidities, such as carotid artery disease, hepatic dysfunction, renal impairment, and pulmonary pathology. These factors, combined with an assessment of the patient’s overall physiologic reserve, help estimate the likelihood of postoperative complications requiring prolonged MCS.

### 2.6. Ability to Rescue/Exit Strategy

While the goal of surgery is to achieve recovery of native cardiac function, particularly in cases of LV dysfunction due to ischemic heart disease or valvular pathology, it is essential to consider exit strategies for patients who may experience refractory heart failure postoperatively. Exit strategies include the potential for heart transplantation or long-term left ventricular assist device (LVAD) support. Patients who are not candidates for either option are considered higher risk, as the absence of a viable exit strategy limits options in the event of prolonged MCS dependency. Candidacy for advanced heart failure therapies thus plays a crucial role in perioperative risk stratification.

### 2.7. Preoperative Imaging

A non-contrast CT chest, abdominal, and pelvis is required to assess the aortic anatomy and length. The primary goal is to confirm that the ascending aorta provides sufficient length to allow for graft anastomosis.

If the aorta is not long enough to accommodate the graft, alternative access sites should be considered, including the axillary or innominate artery for graft placement.

The manufacturer recommends a minimum distance of 7cm from the aortic valve to the graft anastomosis site for Impella 5.5 placement to ensure proper device positioning and function.

### 2.8. Cannulation for Bypass

Aortic Cannulation: The ideal aortic cannulation site is just above the pericardial reflection on the aorta’s lesser curvature. This location is strategically chosen based on its reduced risk of dissection during cannulation, attributed to its position away from the direct path of the blood flow emanating from the LV outflow tract—a contrast to the greater curvature of the aorta. Additionally, this site allows for a low-profile placement of the aortic cannula, minimizing interference with the surgical field and enhancing overall procedural ergonomics.

Venous Cannulation: Right atrial appendage is cannulated with a multistage venous cannula.

### 2.9. Sewing of Chimney Graft on the Ascending Aorta

Following the initiation of cardiopulmonary bypass, a partial aortic clamp is placed to stabilize the aorta. A 10 mm Hemashield graft is then meticulously anastomosed to the ascending aorta in an end-to-side fashion. Once the anastomosis is complete, the graft is temporarily clamped using a Fogarty clamp, allowing for the safe removal of the partial aortic clamp. Anastomosis is rigorously inspected for hemostasis to ensure its integrity. Subsequently, the graft is carefully tunneled through the skin and directed toward the right-side supraclavicular area. This tunneling technique not only secures the graft but also optimizes the positioning for the Impella device, ensuring optimal function and patient comfort.

The planned procedures, like CABG or CABG/AVR, are completed as usual.

Approximately 15 min before the final anastomosis’s completion, the Impella pump’s purge process should be initiated.

During the process of separating from cardiopulmonary bypass, bypass flow is gradually reduced while mechanical ventilation is re-established, and inotropic support is initiated using dual agents at modest doses. Inhaled nitric oxide (iNO) is administered prophylactically to support right ventricular function in patients receiving planned Impella support.

At a bypass flow rate of approximately 4 L/min, the Impella device is inserted with the catheter tip oriented anteriorly. This orientation aligns with the natural curvature of the aorta, which transitions from an anterior to posterior direction as it approaches the aortic root, facilitating smooth passage of the device across the aortic valve. Allowing some degree of native ventricular ejection aids in the opening of the aortic valve, thereby facilitating device placement.

The Impella catheter is advanced under continuous transesophageal echocardiography (TEE) guidance to ensure proper visualization. Once across the aortic valve, the catheter is carefully directed away from the mitral valve apparatus to prevent interference. Prior to activation, it is essential to confirm the absence of stored energy in the device driveline; if present, this can be mitigated by disconnecting and reconnecting the driveline.

Optimal positioning of the Impella involves placing the inlet approximately 5 cm below the aortic valve annulus, with the outlet positioned above the valve, ensuring that the catheter does not interfere with subvalvular structures. The pump should be started at P2–P4 to establish initial flow, with careful monitoring to avoid suction events.

Swan-Ganz catheter monitoring and continuous TEE are employed to assess RV function and intraventricular septal alignment. Pump speeds are adjusted to optimize flow, with ongoing evaluation of the need for additional inotropic support, volume resuscitation, and hemodynamic stability. Throughout this process, it is critical to prevent suction events, which may impose stress on the RV.

Upon complete separation from CPB, the positioning of the Impella device should be reconfirmed using TEE to ensure:

- Accurate alignment within the LV cavity
- Appropriate distance from the mitral valve apparatus
- Optimal pump speed settings to support hemodynamics while minimizing reliance on inotropic agents, assessed through VIS monitoring

Following successful Impella placement and CPB separation, attention should be directed toward achieving surgical hemostasis. Protamine sulfate is administered to reverse heparin anticoagulation, followed by the decannulation procedure according to standard protocols.

### 2.10. Anticoagulation and Impella

The management of anticoagulation in patients supported with MCS, such as the Impella device, involves a dual strategy: systemic anticoagulation and the use of a heparin-based purge solution to maintain pump functionality. The purge solution serves multiple critical roles, including maintaining a positive purge pressure, lubricating the pump’s bearings, and preventing blood entry into the motor, which can lead to thrombosis. Concurrently, intravenous unfractionated heparin is administered to achieve systemic anticoagulation, reducing the risk of thrombus formation within the patient’s circulatory system. Our institutional protocol is to start heparin infusion at 500 units per hour on postoperative day 1 and then aim for a low therapeutic goal of PTT 50–60 until the pump is ready to be removed.

Despite its widespread use, the optimal degree of anticoagulation required to prevent thrombotic complications while minimizing bleeding risks remains an area of active investigation. A recent study involving 114 patients receiving Impella support for cardiogenic shock demonstrated that lower-intensity anticoagulation (targeting an activated partial thromboplastin time [aPTT] of 40–60 s or anti-Xa levels of 0.2–0.3 IU/mL) was associated with reduced rates of both overall and major bleeding compared to standard anticoagulation targets (aPTT 60–80 s or anti-Xa 0.3–0.5 IU/mL), without a significant increase in thrombotic events [17].

In the setting of significant bleeding or coagulopathy, a stepwise de-escalation strategy for anticoagulation is employed to balance the risks of bleeding and thrombosis while maintaining pump function. The Impella system is compatible with a sodium bicarbonate-based purge solution (BBPS), typically prepared with 25 mEq of sodium bicarbonate per 1000 mL of 5% dextrose, which provides an alternative in patients where heparin use is contraindicated.

The de-escalation protocol includes the following steps:

- Discontinuation of systemic heparin while maintaining the heparin-based purge solution. This step helps reduce systemic anticoagulation while preserving local anticoagulation within the device.
- If bleeding or coagulopathy persists, heparin is removed from the purge solution and substituted with BBPS. This transition eliminates all heparin exposure while continuing to maintain pump integrity through purge flow.

This approach allows for individualized anticoagulation management, balancing the competing risks of thrombosis and bleeding in critically ill patients supported with the Impella device. Ongoing research is needed to further refine anticoagulation protocols to optimize outcomes in this high-risk population.

### 2.11. Weaning from MCS

Weaning from perioperative MCS with the Impella device requires a systematic, stepwise approach with close hemodynamic monitoring to ensure a smooth transition to native cardiac function while minimizing the risk of complications. The following protocol is recommended:

Stepwise Reduction in Pump Support: Weaning should be initiated by gradually decreasing the Impella pump performance (P-level) in two-level increments (e.g., from P-6 to P-4, then P-4 to P-2) as cardiac function improves. The P-level should not be reduced below P-2 until just prior to device explantation.

Stabilization Period: Once the P-level is reduced to P-2, it should be maintained at this setting while closely observing the patient’s hemodynamic stability for 12–24 h. If hemodynamics remain stable, the P-level can be decreased to P-1, and then to P-0 at the time of removal.

Echocardiographic Monitoring: Daily echocardiographic assessments are essential to evaluate LV function, the degree of unloading, and proper Impella positioning. TEE or transthoracic echocardiography (TTE) in the long-axis view allows visualization of the aortic valve and Impella inlet area, ensuring optimal device placement

Assessment of RV Function: Swan-Ganz catheterization should be utilized to monitor right ventricular performance and hemodynamics, including pulmonary artery (PA) pressures and central venous pressure (CVP). These measurements help prevent excessive strain on the RV and guide adjustments to maintain balanced biventricular support

Monitoring Perfusion Markers: Daily assessment of perfusion markers is critical, including parameters such as mean arterial pressure (MAP), heart rate, urine output, serum lactate levels, and mixed venous oxygen saturation (SvO_2_). These markers provide valuable insights into tissue perfusion and end-organ function, aiding in decisions regarding further adjustments to Impella support.

Using Metrics Trends for Weaning: The Impella system allows for the use of trend metrics to monitor changes in cardiac output during weaning. It is recommended to enter a new cardiac output measurement each time the P-level is adjusted, ensuring accurate calculations of cardiac power output (CPO)

Explantation Process: Once stable hemodynamics are confirmed at P-0, the Impella catheter can be safely removed following institutional protocols. If using an introducer, ensure the activated clotting time (ACT) is below 150 s before removal to minimize bleeding risk.

### 2.12. Removal of Impella

The removal of the Impella device after successful weaning signifies a transition towards reliance on native cardiac function and the restoration of hemodynamic stability. This process requires meticulous attention to detail, hemodynamic monitoring, and adherence to safety protocols to optimize patient outcomes. If patients exhibit high VIS or signs of poor perfusion, readiness for device removal should be reassessed unless there is a clear exit strategy in place, such as heart transplantation, LVAD implantation, or transition to palliative care.

At the time of explant, the chimney graft frequently contains laminated thrombus. To minimize systemic embolization, the graft should first be controlled proximally and distally, and the device should be withdrawn under echocardiographic and hemodynamic monitoring. Before graft closure, visible thrombus is removed gently with forceps, followed by controlled back-bleeding of the graft to evacuate residual clot burden. This maneuver should be performed in a deliberate and stepwise fashion, avoiding forceful manipulation or uncontrolled forward flushing. Once the graft is judged free of residual thrombus, it is clamped and closed in the standard fashion. When performed with extreme caution, most of the thrombus can be removed while minimizing the risk of embolization.

While Impella removal is often performed in the operating room (OR), bedside explantation can be safely conducted with appropriate resources and continuous monitoring. Bedside removal requires:

- Echocardiographic guidance (preferably TTE or TEE)
- Arterial line monitoring
- Readiness to initiate low-dose vasopressor support (e.g., epinephrine or dobutamine) to maintain hemodynamic stability, if needed

Our preferred approach is device explantation in the operating room, where controlled exposure, direct graft management, and immediate access to surgical intervention provide the safest environment for removal. Bedside explantation in the ICU may be considered in carefully selected hemodynamically stable patients who have been successfully weaned and can undergo removal with continuous echocardiographic and invasive hemodynamic monitoring.

### 2.13. Steps for Bedside Impella Removal

1. Preparation:

Prepare and drape the right supraclavicular region and the Impella device using sterile technique. Position the echocardiographic probe to visualize the Impella crossing the aortic valve. Confirm that the ACT is below 150 s before proceeding.

2. Reduction in Support:

Gradually reduce the Impella pump speed to P-0 to minimize cardiac support while closely monitoring hemodynamics.

3. Device Withdrawal:

Under continuous echocardiographic visualization, gently withdraw the Impella device from the LV into the ascending aorta. Carefully continue device removal through the vascular graft or introducer site, maintaining strict hemodynamic monitoring throughout the procedure.

4. Hemostasis:

After complete device removal, apply a vascular clamp to the graft site to achieve immediate hemostasis. Perform manual compression if necessary, following institutional protocols (typically 40 min of compression).

5. Vascular Graft Closure:

Close the vascular graft using a 5-0 Prolene suture to ensure secure closure and hemostasis. Reinforce the closure with two heavy vascular clips for added security. Irrigate the surgical site thoroughly with Betadine and an antibiotic solution to reduce the risk of infection. Gently guide the vascular graft back into its tunnel, ensuring proper positioning and alignment. Close the deep dermis with 3-0 Vicryl sutures and loosely approximate the skin using a skin stapler to facilitate drainage and minimize tension on the wound.

6. Post-Removal Monitoring:

Continuously monitor hemodynamics, including arterial blood pressure, CVP, and echocardiographic parameters to confirm stable cardiac function after device removal. Evaluate the need for ongoing inotropic support based on VISs and overall clinical status.

This structured approach ensures a safe and effective transition from mechanical support to native cardiac function, minimizing the risk of hemodynamic instability during Impella explantation.

## 3. Results

Planned perioperative Impella 5.5 support was successfully implemented in all patients identified as high risk for PCS based on institutional risk stratification framework. Central Impella placement via a surgically anastomosed ascending aortic chimney graft was successful in all cases. No intraoperative device-related complications, malpositioning, or failures of device deployment were observed.

All patients were successfully separated from cardiopulmonary bypass with Impella support and modest dose inotropic therapy using dual agents. iNO was administered prophylactically to support right ventricular function, and no patients developed intraoperative right ventricular failure requiring escalation of mechanical support.

Postoperative anticoagulation management involved using a dual strategy of systemic unfractionated heparin and a heparin-based purge solution to maintain pump functionality. In addition, a stepwise-deescalation strategy for anticoagulation was in place to balance the risks of bleeding and thrombosis during pump function. No pump thrombosis, purge failure, or device malfunction occurred.

All patients were successfully weaned from MCS and underwent uneventful device explantation either in the OR or at the bedside with echocardiographic guidance. Postoperative survival was 100%, with no in-hospital mortality. All 11 patients demonstrated recovery of native cardiac function sufficient to permit successful weaning from temporary MCS. At two-year follow-up, all patients remained alive.

## 4. Discussion

This early experience suggests that planned perioperative Impella 5.5 support can be implemented safely and effectively in a carefully selected subset of high-risk cardiac surgery patients with severe LV dysfunction undergoing predominantly complex coronary revascularization. In this series of 11 consecutive patients, the mean preoperative LVEF was 17.5% and the mean LV end-diastolic dimension was 6.14 cm, reflecting a cohort with substantial baseline ventricular dysfunction and remodeling. Despite this risk profile, central Impella placement was successful in all cases, all patients were separated from cardiopulmonary bypass with device support, all patients were subsequently weaned from temporary MCS, there was no in-hospital mortality, and all patients remained alive at two-year follow-up. Taken together, these findings support the feasibility of a planned, protocol-driven Impella strategy in selected patients at heightened risk for postcardiotomy shock.

The most important message from this series is not that Impella should be used broadly in all patients with low LVEF undergoing cardiac surgery, but rather that outcomes may be favorable when the device is used proactively in a narrow population selected on the basis of operative intent, myocardial reserve, anticipated postoperative course, and the presence of a viable rescue strategy. In our cohort, the operations were not routine low-risk CABG cases; many involved complex multivessel revascularization and selected concomitant procedures, with a mean STS predicted mortality of 5.49%. These characteristics likely identify patients in whom the physiologic stress of cardiopulmonary bypass and reperfusion could overwhelm limited ventricular reserve and in whom prophylactic unloading and circulatory support may be most valuable. Thus, our results should be interpreted as support for a selective rather than indiscriminate use paradigm.

The observed postoperative course is also important to interpret. All patients were able to come off bypass with Impella support and modest dual-agent inotropy, no patient required escalation for intraoperative right ventricular failure, no pump thrombosis, purge failure, or device malfunction occurred, and all patients underwent successful explantation either in the operating room or at the bedside. These findings suggest that, in addition to device choice, the success of this strategy likely depends on a reproducible perioperative workflow that includes standardized implantation, echocardiographic confirmation of position, vigilant right ventricular assessment, structured anticoagulation management, and protocolized weaning. In other words, the favorable outcomes in this series should not be attributed to the device alone, but to the combination of patient selection and disciplined perioperative management.

Our findings are directionally consistent with prior observational reports suggesting that prophylactic Impella support may improve perioperative stability in patients with severe LV dysfunction undergoing high-risk cardiac surgery. However, the present study adds a practical technical perspective by describing a central chimney-graft strategy and a perioperative management framework that can be replicated in experienced centers. The absence of perioperative mortality or failure to wean in this series compares favorably with the historically poor outcomes associated with postcardiotomy shock and with previously reported observational Impella series, while remaining hypothesis-generating rather than definitive.

Importantly, this approach should not be interpreted as universally applicable to all centers managing high-risk CABG patients with severe LV dysfunction. Successful implementation of a planned perioperative Impella program depends on multiple institutional factors, including surgeon familiarity with temporary MCS, echocardiographic expertise, ICU experience with postoperative device management, and the availability of advanced heart failure rescue pathways when needed. In addition, alternative support strategies remain relevant in this population. For patients with established postcardiotomy shock, severe hypoxemia, or biventricular failure, combined approaches such as VA-ECMO with LV unloading using either IABP or Impella may be more appropriate, while selected patients may also require combined IABP and Impella support depending on the hemodynamic goals. Each configuration has advantages and disadvantages, and no single device should be viewed as a universal solution. Broader adoption of planned perioperative Impella support will also depend not only on clinical outcomes but on institutional resource availability and future analyses of cost-effectiveness and value in this highly selected population.

Although survival of all patients at two years is reassuring, follow-up of this length is still insufficient to fully characterize the long-term implications of a planned perioperative Impella strategy. This is particularly relevant because the present study is intended to support a technique and treatment pathway rather than simply report procedural success. Longer follow-up would be helpful to determine whether the early perioperative benefit translates into durable clinical improvement, sustained myocardial recovery, freedom from heart failure hospitalization, and acceptable late survival. Extended follow-up would also better define whether there are delayed complications related to the graft site, vascular reconstruction, recurrent heart failure, or progression to durable LVAD or transplantation in this population. Accordingly, our current results should be interpreted as strong early and mid-term feasibility data rather than evidence of definitive long-term benefit.

This study has several limitations. It is a retrospective, single-center experience describing only 11 patients and lacks a contemporaneous comparison group. As such, it is subject to substantial selection bias and cannot establish superiority over conventional management or over other MCS strategies. In addition, although the cohort had severe LV systolic dysfunction, more granular reporting of myocardial reserve variables, including wall thickness, regional wall motion abnormalities, and patient-level application of the selection framework, would enhance interpretability and reproducibility in future studies.

In summary, this early Johns Hopkins experience supports the concept that planned perioperative Impella 5.5 support may be a useful adjunct in a highly selected subgroup of patients with severe LV dysfunction undergoing high-risk CABG-based surgery. The signal from this series is strongest for feasibility, successful separation from bypass, successful weaning from temporary support, and excellent short- to mid-term survival. The next step is not broader unselected adoption, but prospective evaluation with clearer selection variables, standardized outcome reporting, and longer follow-up to determine whether the early perioperative advantages of this strategy translate into durable clinical benefit.

## 5. Conclusions

Planned perioperative Impella 5.5 support is a feasible and safe strategy for high-risk cardiac surgery patients with severe LV dysfunction. Our early experience demonstrates excellent short- and mid-term survival with successful weaning from temporary MCS. However, larger studies are needed to validate these findings and define optimal patient selection.

## Figures and Tables

**Table 1:** Comparison of MCS hemodynamics.

	Mechanism	LV Unloading	Afterload	Myocardial Oxygen Consumption (MVO_2_)	Coronary Perfusion	Complications
Vasoactive agents						
Pressors		–	↑	↑	–	End-organ damage and peripheral malperfusion
Inotropes		–	↓	↑	–	
MCS Devices						
IABP	Pneumatic	+	↓	↓	↑	Limb/spinal cord ischemia. Access complications, aortic dissection
VA-ECMO	Centrifugal	–	↑↑↑	–	–	Stroke/Access complications
Impella	Microaxial	++	↓	↓	↑	Right Ventricular (RV) failure

Symbols indicate relative directional effect or magnitude: ++, marked effect; +, modest effect; –, absent or unfavorable effect; ↑, increase; ↓, decrease.

**Table 2:** Patient Summary.

Procedure Summary	Age/Sex	STS Operative Mortality (%)	Preop Echo, LVEF (%)	Preop Echo, LVDD (cm)
CABG×5	80/M	11.5	5–10	6.1
CABG×2	67/M	2.45	10–15	6.8
CABG×2	56/M	1.94	15–20	5
CABG×4, L CEA	63/M	8.33	25–30	5.8
CABG×3	71/M	2.89	20	6.2
CABG×3, PVI, LAA	46/M	5.6	20–25	7.8
CABG×2, MVR	59/M	2.92	20	7.2
CABG×4	72/M	3.14	10–15	5.9
CABG×3	60/M	7.33	20–25	6.9
CABG×3	61/F	8.79	15–20	4.28
CABG×1, AAR	58/M	2.39	10–15	5.52
Mean ± SD	63 ± 9.18	5.49 ± 3.32	17.5 ± 5.81	6.14 ± 1.01

**Table 3:** Intraoperative Data.

Procedure Summary	Age/Sex	Total CPB Time (min)	Total Cross-Clamp Time (min)	Initial VIS
CABG×5	80/M	141	80	7
CABG×2	67/M	112	54	10
CABG×2	56/M	114	68	14
CABG×4, L CEA	63/M	137	87	6
CABG×3	71/M	160	81	5
CABG×3, PVI, LAA	46/M	158	71	6
CABG×2, MVR	59/M	286	207	15
CABG×4	72/M	165	95	15
CABG×3	60/M	113	66	8
CABG×3	61/F	145	99	7
CABG×1, AAR	58/M	89	48	8
Mean ± SD	63 ± 9.18	147.27 ± 50.74	86.9 ± 43.07	9.30 ± 3.95

**Table 4:** Postoperative Data.

Procedure Summary	Age/Sex	VIS 6 h	VIS 24 h	VIS 48 h	Length of Impella Support (h)	Delta Creatinine Day 3	Maximum Lactate	Time to Lactate Clearance (h)
CABG×5	80/M	7	7	7	72	−0.2	7.1	10
CABG×2	67/M	13	9	7	48	1.7	9.5	18
CABG×2	56/M	14	26	6	96	−0.2	9.7	12
CABG×4, L CEA	63/M	4	2	3	48	0.2	5.4	10
CABG×3	71/M	5	10	6	96	−0.1	7.6	11
CABG×3, PVI, LAA	46/M	4	3	0	48	0.9	13.6	22
CABG×2, MVR	59/M	12	9	5	264	−0.1	5.4	8
CABG×4	72/M	15	15	12	96	−0.1	3.9	15
CABG×3	60/M	10	2	3	96	0.3	6.1	9
CABG×3	61/F	7	1	5	192	−0.3	3.1	16
CABG×1, AAR	58/M	6	5	0	168	0.1	13.2	17
Mean ± SD	63 ± 9.18	9.19 ± 3.95	8.40 ± 7.63	5.40 ± 3.17	105.06 ± 69.93	0.21 ± 0.63	8.65 ± 3.313	13.45 ± 4.44

**Table 5:** Patient length of stay, morbidity and mortality.

Procedure Summary	Age/Sex	Postop ICU LOS (Days)	Postop Total LOS (Days)	Major Postop Morbidities	30-Day Mortality	60-Day Mortality	60-Day Re-Admission
CABG×5	80/M	5	11		No	No	No
CABG×2	67/M	10	11		No	No	No
CABG×2	56/M	6	7	DSWI	No	No	No (sternal wound recon with PRS)
CABG×4, L CEA	63/M	6	22	CVA	No	No	No
CABG×3	71/M	8	11		No	No	No
CABG×3, PVI, LAA	46/M	9	10		No	No	No
CABG×2, MVR	59/M	21	27		No	No	Yes (arrythmia)
CABG×4	72/M	20	25		No	No	No
CABG×3	60/M	7	12	RTOR–bleeding	No	No	Yes (pneumonia)
CABG×3	61/F	9	13		No	No	No
CABG×1, AAR	58/M	8	9		No	No	No
Mean ± SD	63 ± 9.18	9.91 ± 5.71	14.90 ± 7.02	27%	0%	0%	18%

## Data Availability

The raw data supporting the conclusions of this article will be made available by the authors on request.

## Abbreviations

The following abbreviations are used in this technical note:

PCS	Postcardiotomy shock
LVEF	Left Ventricular Ejection Fraction
MCS	Mechanical Circulatory Support
IABP	Intra-Aortic Balloon Pump
VA-ECMO	Venoarterial Extracorporeal Membrane Oxygenation
LV	Left Ventricle/Ventricular
CABG	Coronary Artery Bypass Graft
VIS	Vasoactive Inotropic Scores
LVAD	Left Ventricular Assist Device
iNO	Inhaled Nitric Oxide
TEE	Transesophageal Echocardiography
BBPS	Bicarbonate-Based Purge Solution
TTE	Transthoracic Echocardiography
RV	Right Ventricle/Ventricular
CVP	Central Venous Pressure
CPO	Cardiac Power Output
ACT	Activated Clotting Time
OR	Operating Room
